# Assessment of daily variation in pelvic anatomy in women with and without pelvic organ prolapse

**DOI:** 10.1007/s00192-023-05550-0

**Published:** 2023-05-05

**Authors:** Annemarie van der Steen, Lisan M. Morsinkhof, Luyun Chen, Mirjam de Vries, Frank F. J. Simonis, Anique T. M. Grob

**Affiliations:** 1 Multi Modality Medical Imaging (M3I), TechMed Centre, University of Twente, Drienerlolaan 5, Enschede, The Netherlands; 2 Department of Gynecology, Ziekenhuisgroep Twente, Hengelo/Almelo, The Netherlands; 3 Magnetic Detection and Imaging (MD&I), TechMed Centre, University of Twente, Enschede, The Netherlands; 4 Department of Obstetrics and Gynecology, University of Michigan, Ann Arbor, MI USA

**Keywords:** Magnetic resonance imaging, Pelvic inclination correction system, Pelvic organ prolapse, Levator plate, Upright, Daily variation

## Abstract

**Introduction and hypothesis:**

Symptoms of pelvic organ prolapse (POP) can affect quality of life and are reported to progress during the day, although this was never objectified. The aim of this study is to determine whether the pelvic anatomy changes during the day using upright magnetic resonance imaging (MRI), in POP patients and asymptomatic women.

**Methods:**

In this prospective study 15 POP patients and 45 asymptomatic women were included. Upright MRI scans were obtained three times per day. The distances from the lowest points of the bladder and cervix to a standardized reference (pelvic inclination correction system) line were determined. A principal component analysis was performed on the levator plate (LP) shape. Statistical differences between time points and the groups were determined for the bladder, cervix, and LP shape.

**Results:**

For all women a significant decrease in bladder and cervix height of −0.2 cm (*p*<0.001) was seen between morning/midday and afternoon scans. A significant difference in bladder descent during the day between POP patients and asymptomatic women was found (*p*=0.004). Individual differences in bladder position in the POP group of up to 2.2 cm between the morning and afternoon scan were reported. There was a significant difference in LP shape (*p*<0.001) between the groups but there were no significant changes during the day.

**Conclusions:**

This study found no clinically relevant pelvic anatomy changes during the day. Still, on an individual level differences can be large, so repeating clinical examination at the end of the day can be recommended in patients when anamnesis and physical examination do not match.

## Introduction

Pelvic organ prolapse (POP) is a common condition affecting up to 50% of postmenopausal women [1]. It is defined as a downward displacement of one or more of the pelvic organs such as the bladder, the uterus or vaginal vault, and the rectum [2]. POP is a benign condition, but it can affect quality of life by causing symptoms such as vaginal bulging, pelvic pressure, and incomplete emptying of the bladder or rectum. In addition, many women describe their symptoms worsening during the day [3]. It is known that the cardinal and uterosacral ligaments have a viscoelastic property, which means that loading of these ligaments over time may cause lengthening, resulting in a descent of the pelvic organs during the day. Cheng et al. showed that straightening and lengthening of the cardinal ligaments correlates strongly with prolapse [4]. We hypothesize that the worsening of POP symptoms during the day might be coupled with an increase in the extent of prolapse during the day, caused by people being in upright position for a considerable time [5]. Therefore, the moment of diagnosis may influence the way in which clinicians should interpret POP measurement, and possibly even treatment decisions.

In the literature contradictory results have been reported regarding differences in prolapse severity during the day. On the one hand research using daily symptom diaries showed that the severity of prolapse symptoms varies during the day and is most severe in the evening [3]. On the other hand standard gynecological clinical examination (POP-Q) in patients with prolapse did not show a (clinically relevant) difference between measurements during the day [6, 7]. This might be caused by the fact that the examinations are being performed in the dorsal lithotomy position, as is most common in clinical practice. Odegaard et al. [6] concluded that the Valsalva maneuver, which is used during POP-Q examination, should be sufficient to demonstrate the maximal prolapse that women experience at any moment of the day. Therefore, we hypothesize that the supine POP-Q examination might not be representative of the natural descent of the prolapsed organs, which happens during the day.

Imaging techniques such as magnetic resonance imaging (MRI) can be a useful addition to physical examination as it can give information on multicompartment prolapse under standardized conditions. Furthermore, Grob et al. [8] showed that prolapse extent is underestimated using MRI with the patient in a supine straining position compared with the upright position at rest. This makes the upright MRI scan superior to the supine POP-Q measurements when looking at pelvic anatomy changes during the day.

Apart from the position of the organs a second pelvic anatomy parameter, the levator plate (LP) shape, is of interest when studying changes during the day. The LP is the shelf on which the pelvic organs rest, and recently its angle and shape were found to be associated with prolapse and recurrences after surgery [9, 10]. Based on these findings we hypothesize that the LP shape might also change during the day, in line with our hypothesis on pelvic organ descent.

The primary objective of this study is to determine if organ position and LP shape vary during the day, evaluated using magnetic resonance (MR) scans in an upright position. Because it remains unknown how the pelvic anatomy changes during the day in asymptomatic women, and whether this differs at different stages of life, the secondary objective is to determine if this daily variation varies between POP patients and asymptomatic women depending on vaginal delivery and menopausal status.

## Materials and methods

### Population

In this prospective study, 60 women were included for analysis of daily variation in bladder and cervix height and levator plate shape. Fifteen POP patients were selected from the gynecology department of the Ziekenhuisgroep Twente (ZGT) hospital in Hengelo, the Netherlands, and 45 asymptomatic women enrolled via flyers. The study was approved by the medical ethics committee and registered as NL74061.091.20. All women gave written informed consent.

Women were included in separate groups of 15 participants, with different inclusion criteria:

1. Nulliparous, pre-menopausal status, no symptoms related to prolapse or incontinence in history (nulliparous)
2. Parous, pre-menopausal status, no symptoms related to prolapse or incontinence in history (parous)
3. Parous, post-menopausal status, no symptoms related to prolapse or incontinence in history (postmenopausal)
4. Parous, post-menopausal status, symptomatic prolapse, pelvic organ prolapse quantification (POP-Q) stage ≥2, prolapse of the anterior and/or middle compartment (POP)

All women were 18 years or older and were excluded if they were not able to stand for 20 min without assistance, were unable to refrain from lying down from 8 am to 6 pm, were not eligible to undergo an MR scan in response to an MR safety checklist, or had a jeans size ≥52 (EU) or 22 (USA), because of the limited coil circumference.

### MRI examination

Magnetic resonance scans of the women in an upright position were acquired three times during 1 day: in the morning (8–10 am), midday (12 am to 2 pm), and afternoon (4–6 pm). The participants were not allowed to drink for 1 h before the scan and had to empty their bladder within 15 min before the scan. During the time between the scans, the women were encouraged to mimic their normal daily activity as much as possible. They were not allowed to lie down.

A tiltable 0.25 T MR scanner (G-Scan Brio; Esaote, Genoa, Italy) was used for MR acquisition, with a dedicated multichannel spine coil. A 3D balanced steady-state free precession (bSSFP) sequence was acquired in an upright patient position (echo time/repetition time: 4/8 ms, flip angle: 60°, reconstructed resolution: 0.49 × 0.49 × 0.49 mm^3^, field of view: 250 × 250 × 122 mm^3^ or 250 × 250 × 160 mm^3^, acquisition matrix 124 × 124 × 100, number of signal averages 3, scan time: ±5 min).

### Image analysis

**Fig. 1:**
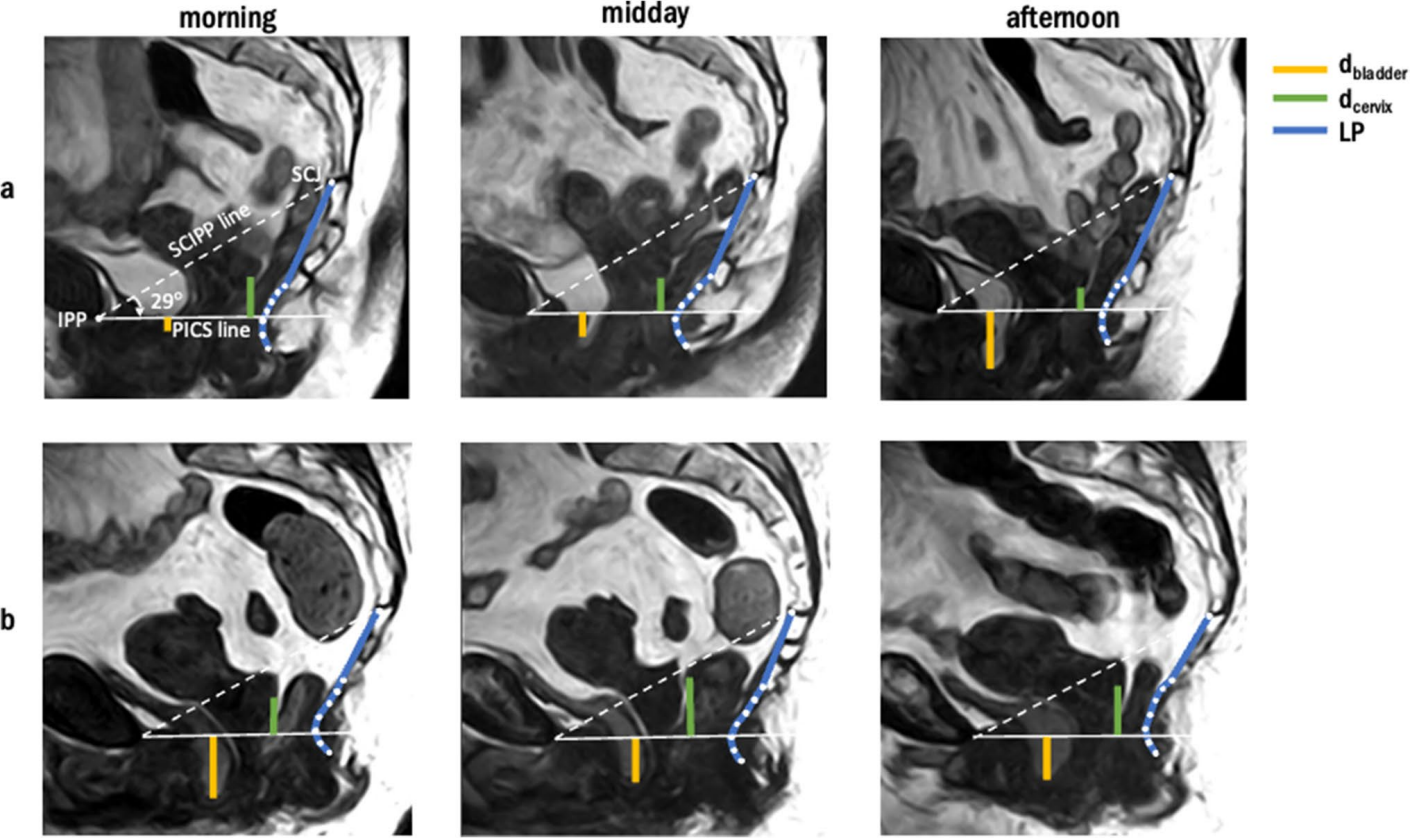
Sagittal magnetic resonance images of two pelvic organ prolapse (*POP*) patients at the three moments during the day, on which distance from lowest point of the bladder to the pelvic inclination correction system (*PICS*) line (d_bladder_), distance from the posterior point of the cervix to the PICS line (d_cervix_), and the levator plate (*LP*) were visualized. **a** The bladder descended from 0.6 cm below the pelvic inclination correction system (*PICS*) line in the morning to 2.8 cm below the PICS line in the afternoon. The cervix descended from 1.4 to 1.0 cm above the PICS line. **b** The bladder ascended from 3.2 to 2.4 cm below the PICS line, the cervix ascended from 1.6 to 2.2 cm above the PICS line. *IPP* inferior pubic point, *SCJ* sacrococcygeal joint, *SCIPP* sacrococcygeal to inferior pubic point line

The software MATLAB 2021a (MathWorks, Natick, MA, USA) was used to annotate the inferior pubic point (IPP), sacrococcygeal joint (SCJ), the lowest point of the bladder, and the posterior part of the cervix. Based on these annotations the bladder and cervix height were determined, defined as the perpendicular distance from the lowest point of the bladder and the posterior part of the cervix to the upright pelvic inclination correction system (PICS) line, d_bladder_ and d_cervix_ respectively (Fig. 1). The upright PICS line is based on a fixed clockwise rotation of 29° with respect to the sacrococcygeal–inferior pubic point (SCIPP) line, and was previously validated as a reference system to standardize the prolapse quantification on upright MRI [11]. In this study we did not assess rectocele, because the posterior vaginal wall and rectum were poorly visualized on the MRI when not using rectal contrast medium.

According to a previously published method [10], LP annotations on the upright scans were performed using ImageJ software (Fig. 1) [12] and based on those annotations, statistical shape analysis was executed using a principal component analysis method in a custom Python program. Subsequently, the mean LP shape over all women was calculated for each time point during the day. The two principal components (PCs) representing the most significant independent shape variations (PC1 and PC2) were selected for further analysis. PC1 and PC2 scores were calculated for each scan per time point, by analyzing the difference between the scan-specific LP shape and the mean LP shape of the corresponding time point.

### Statistical analysis

Statistical analyses were performed using SPSS version 28.0.1.0 (SPSS, Chicago, IL, USA). Normality of the data was assessed by visual inspection of histograms. Demographic differences between the postmenopausal group and the POP group were analyzed using the Wilcoxon signed-rank test. The mixed model repeated measures analysis was performed to evaluate differences between d_bladder_ and d_cervix_ of the morning, midday, and afternoon scans. For the statistical analysis of the PCA of the LP shape a two-way ANOVA was performed to determine whether there was any difference in groups, times, as well as interaction between the groups and the time.

## Results

### Demographics

In total, of 59 women three scans were acquired. In one woman only two scans were acquired because she fainted during the last scan. Scans of two participants (one in the postmenopausal and one in the POP group) were discarded for cervix analysis because the posterior part of the cervix was not distinguishable. An overview of the demographics is presented in Table 1. Women of the POP group were significantly older than the women in the postmenopausal group (*p*=0.02), with a median difference of 3 years.

**Table 1:** Demographic data of the study population

	Total (*n*=60)	Nulliparous (*n*=15)	Parous (*n*=15)	Postmenopausal (*n*=15)	POP (*n*=15)
Age (years)	50 (28–61)	23 (21–25)	39 (36–45)	59 (54–61)	62 (59–70)
BMI (kg/m^2^)	25 (23–27)	22 (20–25)	25 (23–28)	26 (24–29)	25 (23–27)
Parity	2 (0–2)	0 (0–0)	2 (2–2)	2 (2–3)	2 (2–3)

Data are presented as median (interquartile range)

*BMI* body mass index, *POP* pelvic organ prolapse

### Outcomes

**Fig. 2:**
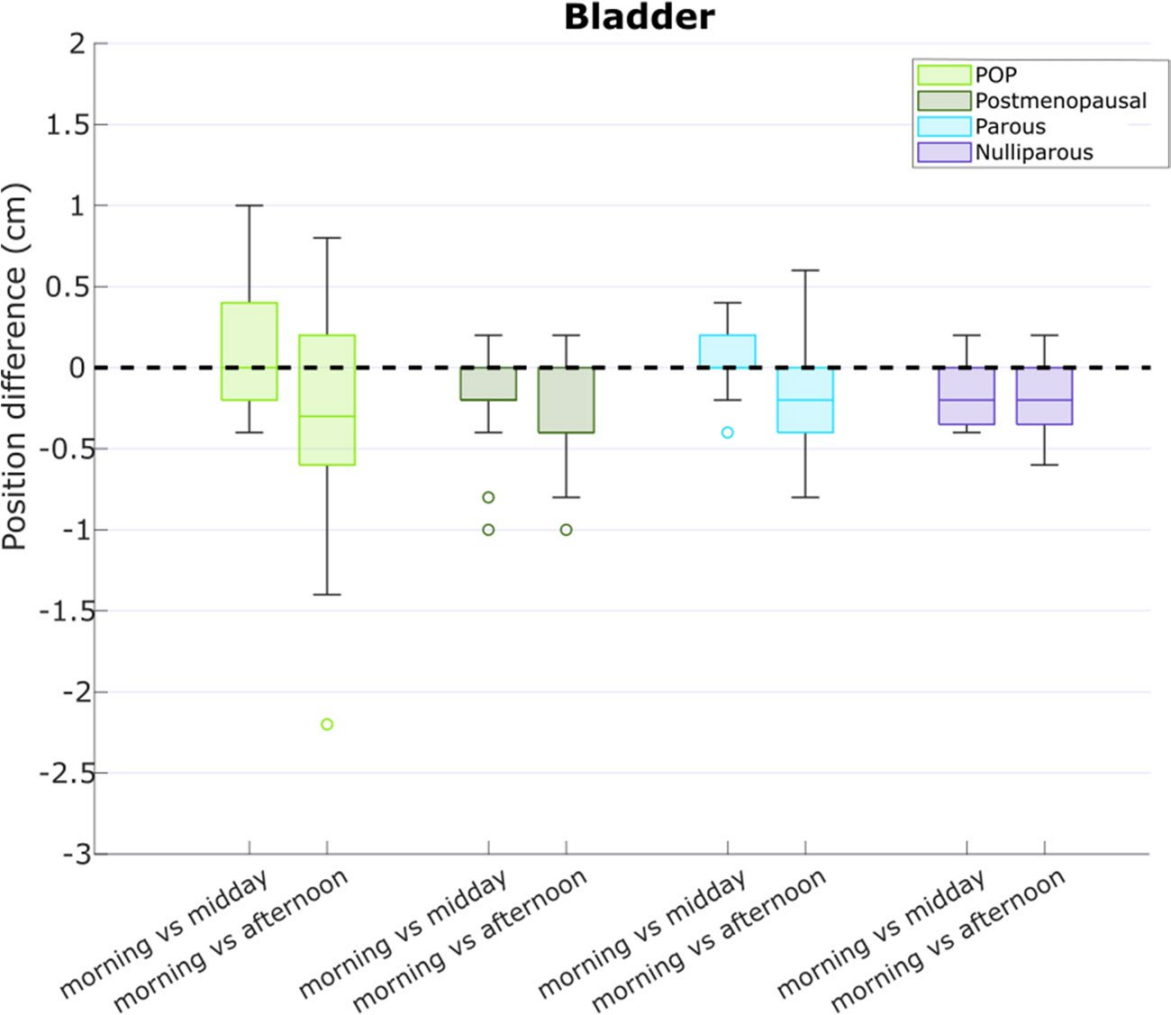
Bladder height differences between time points during the day, for the different subgroups. The median (interquartile range) difference between the morning and afternoon measurements was −0.2 (0.0, −0.4) cm, but this difference can be as large as −2.2 cm for individual subjects. *POP* pelvic organ prolapse

Analyzing bladder height, taking all women into account, there was a significant decrease during the day, with a median (interquartile range (IQR)) difference of −0.2 (0.0, −0.4) cm in the afternoon compared with the morning (*p*<0.001; Fig. 2). Pairwise comparison revealed a significant descent of the bladder between morning–afternoon (*p*<0.001) and midday–afternoon (*p*<0.001), but not between morning and midday (*p*=0.39). The bladder descent was significantly larger in the POP group compared with all asymptomatic groups (*p*<0.001 for all three asymptomatic groups), and in the postmenopausal group compared with the nulliparous group (*p*=0.001). The median (IQR) difference in bladder height between the morning and afternoon measurements of the POP group was −0.3 (−0.8, 0.2) cm.

**Fig. 3:**
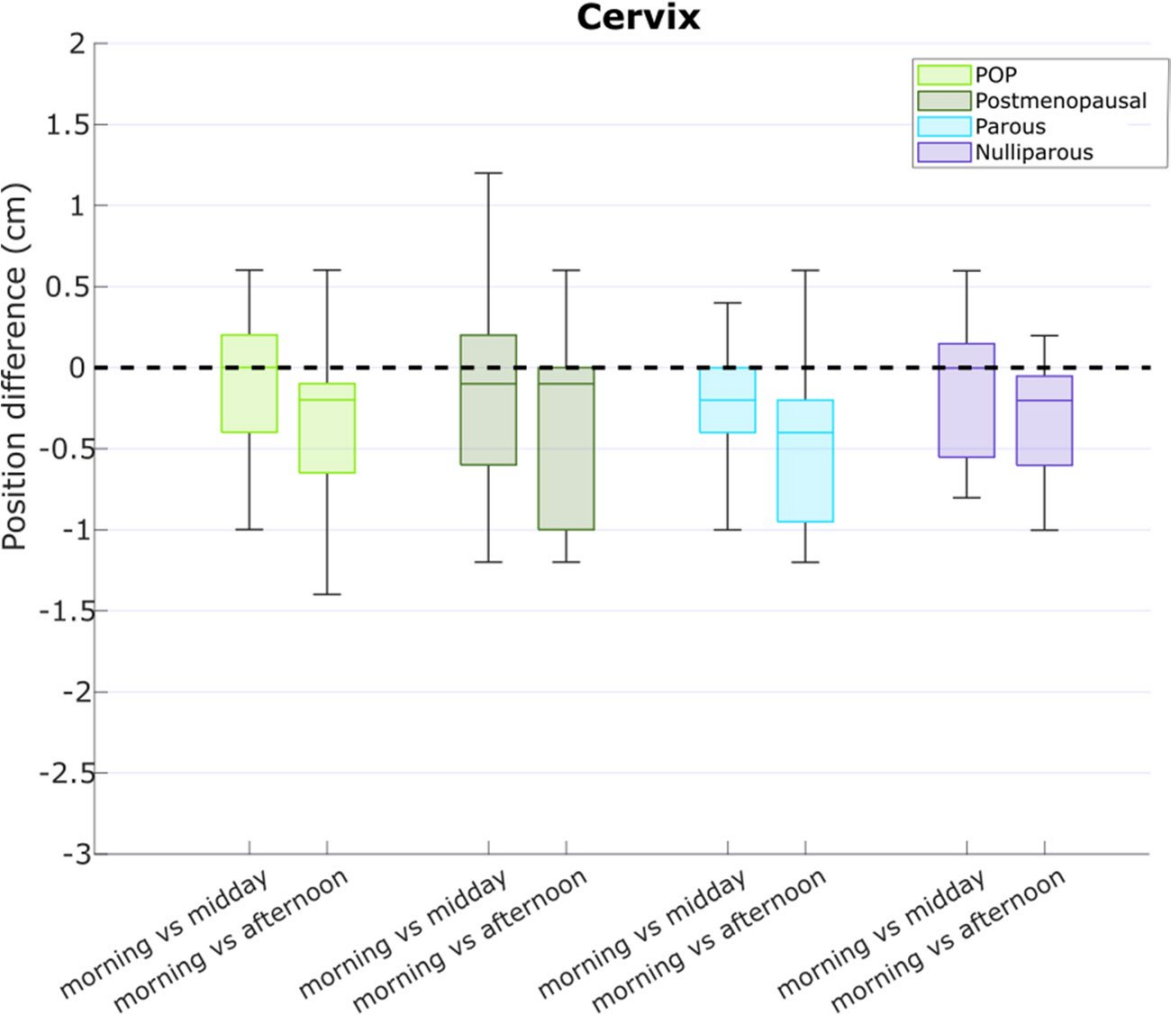
Cervix height differences between time points during the day, for the different subgroups. The median (interquartile range) difference between the morning and afternoon measurements was −0.2 (0.0, −0.8) cm, but this difference can be as large as −1.4 cm for individual subjects. *POP* pelvic organ prolapse

The cervix height significantly decreases during the day (*p*<0.001; Fig. 3) in all women, with a median (IQR) difference of −0.2 (0.0, −0.8) cm in the afternoon compared with the morning. Pairwise comparison revealed a significant difference between morning–afternoon (*p*<0.001) and midday–afternoon (*p*<0.001), but not between morning–midday (*p*=0.11). No significant difference in daily variation between the different groups was found (*p*=0.97).

The median difference between the different time points is small for both the bladder and cervix height, with reported outliers up to −2.2 cm for the bladder and −1.4 cm for the cervix extent between the morning and afternoon measurements (Figs. 1, 2).

**Fig. 4:**
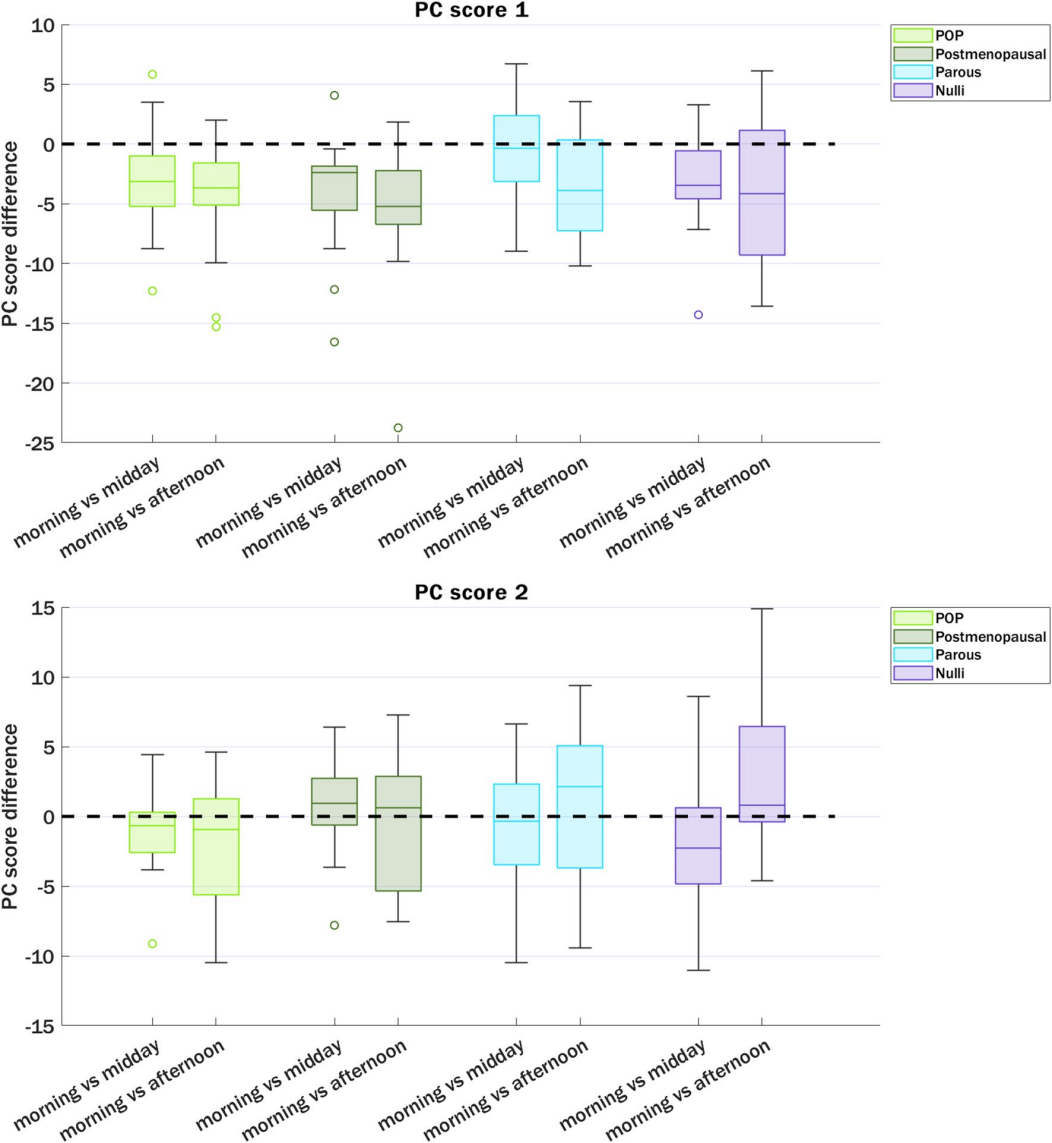
Principal component (*PC*)1 and PC2 score differences between time points during the day, for the different subgroups. There were no significant differences in median (interquartile range) between the morning and afternoon measurement scores for all groups. *POP* pelvic organ prolapse

**Fig. 5:**
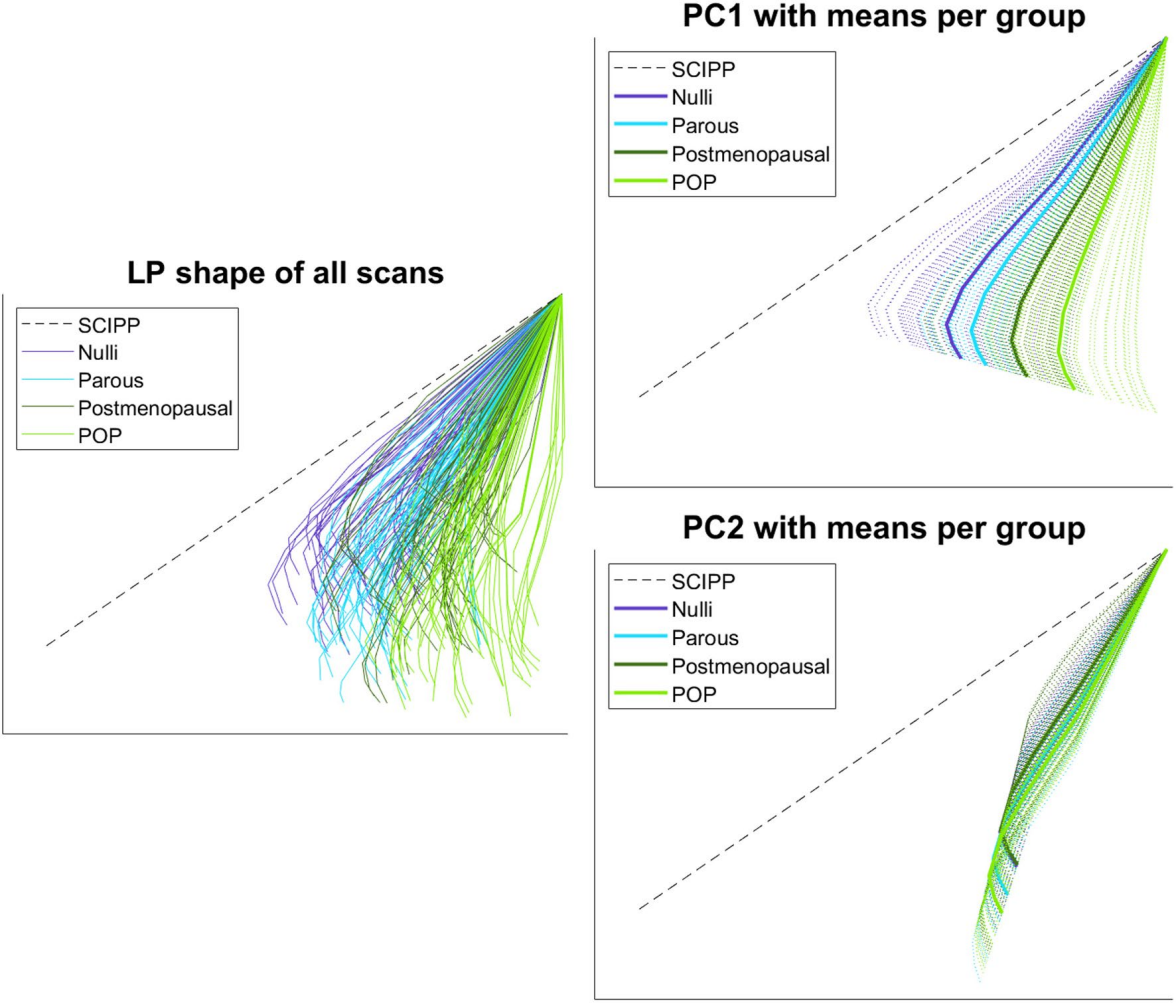
Levator plate shape in the 45 asymptomatic women divided into three groups and 15 pelvic organ prolapse (*POP*) patients. Principal component (*PC*)1 accounts for 61% of shape variation and PC2 accounts for 33%. *LP* levator plate, *SCIPP* sacrococcygeal–inferior pubic point

For the LP shape two main PCs were identified with shape variations: PC1 accounted for 61% of shape variation and PC2 for 33%. For PC1 an interaction between group and time of day could not be demonstrated, *F*(6, 165)=0.041, *p* = 1.00. There was a significant difference in mean PC1 scores between the groups (*p*<0.001) but not for the different time points (*p*=0.306). In the multiple comparisons there was a significant difference in mean PC1 scores between all the groups, but not for all time points (Fig. 3). For PC2 an interaction between group and time of day could not be demonstrated, *F*(6, 165)=0.158, *p*=0.98. There was a significant difference in mean PC2 scores between the groups (*p*<0.001) but not for the different time points (*p*=0.911). In the multiple comparisons there was no significant difference in mean PC2 for nulli–postmenopausal (*p*=0.995), POP–parous (*p*=0.152), and for all the timepoints. For all the other comparisons the mean differences were significant. The variation captured by these PCs was visually investigated and revealed a cranio-caudal and anterior–posterior shape variation for PC1. The LP shape seems to rotate dorsally around the sacrococcygeal joint with the most dorsal rotation in the POP group, then the postmenopausal group, the parous group, and the nulli group (Fig. 4). PC2 captures mostly the length shape variation; visual difference was minimal between groups (Fig. 5).

## Discussion

The current study found a significant descent of the bladder and cervix height for all women during the day. The daily variation in bladder height was found to be significantly larger in the POP group than in the asymptomatic groups and in the postmenopausal group compared with the nulliparous group. However, both the median variations in bladder and cervix height during the day, and the difference in daily variation between groups were relatively small compared with the smallest difference between the two POP-Q stages, which is equal to 1 cm [2]. Although we hypothesized that progression of prolapse symptoms during the day might be due to the natural descent of organs in an upright position, daily variation in bladder and cervix height was not found to be clinically relevant in asymptomatic women and POP patients. Additionally, this study found no statistical difference in LP shape during the day, neither for POP patients nor for asymptomatic women.

Our findings using upright MRI were in line with those of previous publications, in which no daily variation was found using physical examination in a supine position [6, 7]. This means that no explanation for the increase in POP symptoms during the day was found. Comparing our results concerning the LP shape with those of previous research was difficult, because only supine scans were performed. We did, however, establish a significant difference in LP shape between POP patients and asymptomatic women, which is in line with its reported association with prolapse and prolapse recurrence after surgery [9, 10].

Interestingly, on an individual level there were exceptions in which the variation between two measurements of the bladder and cervix were larger than 1 cm, mainly in the postmenopausal and POP groups. This difference could be as large as 2.2 cm in the case of the bladder height (Fig. 1a). In these cases, the measured extent of prolapse was considerably influenced by the moment of prolapse examination, which may bias therapy planning by underestimation of the prolapse when examined in the morning. Therefore, it is recommended to repeat prolapse examination at another moment during the day if the anatomical findings of the examination are not in line with the described symptoms.

It is additionally interesting to note that not only a descent of the organs but also an ascent during the day was found in certain women. A possible explanation for this is that the bladder and cervix height were not solely influenced by the amount of time in an upright position but also by other factors that vary during the day that were not controlled in this study. One example has been reported before by Hassan et al. [13], who found a decrease in the sensitivity of cystocele and uterine prolapse detection during MR defecography after rectal filling, which can be the cause of a higher organ position. A full bladder is also reported to be associated with underestimation of the severity of prolapse [14–16].

Strengths of our study were its prospective design, which included POP patients as well as asymptomatic women at different stages of life. This enabled comparison between those groups, and thereby helped to determine which were normal anatomical changes during life, and which were specific to POP patients. Furthermore, we included direct organ measurements by means of pelvic organ heights, but also the organ support mechanism by means of the LP shape, which gave a more in-depth analysis of the pelvic anatomy.

A limitation of this study was that in the POP group we included patients with prolapse stage ≥2 of the bladder, or bladder combined with uterus, and we performed the measurements of the anatomical parameters in all these patients without differentiating between these types of prolapse. However, looking into the results all patients had a bladder prolapse of at least POP-Q stage 2, whereas only 27% of the patients also had a stage 2 uterus prolapse. This resembled clinical practice, where multicompartment prolapse is present in some of the patients and might explain why there was no difference between the groups for cervical descent during the day.

We limited the number of factors that were standardized for POP patients and asymptomatic women in this study. For example, daily activity, bladder filling, and rectal filling were not standardized, whereas they might have influenced the measurements. On the other hand, we asked women to match their activities on the scanning day to their normal daily activity and all women were asked to empty their bladder 15 min before the scan and not drink in the hour before. In this way we could mimic the women’s daily practice, in which they would normally experience their prolapse symptoms, as much as possible. An option for future research could be to perform the MRI scans with standardized bladder filling and activities during the day of scanning.

Based on our findings we can conclude that on average in prolapse patients as well as in asymptomatic women there is no clinically relevant variation in bladder height, cervix height, and LP shape during the day. However, on an individual level there can be a larger variation in organ height of up to 2.2 cm. We therefore recommend repeating prolapse examination at different moments in case the measured parameters are not in agreement with the symptoms of a patient.

## Data Availability

The data that support the findings of this study are available from the corresponding author, AS, upon reasonable request.
